# The Finite Size Effects and Two-State Paradigm of Protein Folding

**DOI:** 10.3390/ijms22042184

**Published:** 2021-02-22

**Authors:** Artem Badasyan, Matjaz Valant, Jože Grdadolnik, Vladimir N. Uversky

**Affiliations:** 1 Materials Research Laboratory, University of Nova Gorica, Vipavska 13, SI-5000 Nova Gorica, Slovenia; matjaz.valant@ung.si; 2 Institute of Fundamental and Frontier Sciences, University of Electronic Science and Technology of China, Chengdu 610054, China; 3 National Institute of Chemistry, Hajdrihova 19, SI-1000 Ljubljana, Slovenia; joze.grdadolnik@ki.si; 4 Department of Molecular Medicine, USF Health Byrd Alzheimer’s Research Institute, Morsani College of Medicine, University of South Florida, 12901 Bruce B. Downs Blvd., MDC07, Tampa, FL 33612, USA; vuversky@health.usf.edu

**Keywords:** protein folding, two-state model, size scaling, thermodynamic cooperativity

## Abstract

The coil to globule transition of the polypeptide chain is the physical phenomenon behind the folding of globular proteins. Globular proteins with a single domain usually consist of about 30 to 100 amino acid residues, and this finite size extends the transition interval of the coil-globule phase transition. Based on the pedantic derivation of the two-state model, we introduce the number of amino acid residues of a polypeptide chain as a parameter in the expressions for two cooperativity measures and reveal their physical significance. We conclude that the $k_{2}$ measure, defined as the ratio of van ’t Hoff and calorimetric enthalpy is related to the degeneracy of the denatured state and describes the number of cooperative units involved in the transition; additionally, it is found that the widely discussed $k_{2}=1$ is just the necessary condition to classify the protein as the two-state folder. We also find that $\Omega_{c}$, a quantity not limited from above and growing with system size, is simply proportional to the square of the transition interval. This fact allows us to perform the classical size scaling analysis of the coil-globule phase transition. Moreover, these two measures are shown to describe different characteristics of protein folding.

## 1. Introduction

From the point of view of polymer physics, the folding of a protein is similar to the coil-globule transition of a short polypeptide chain [1]. The coil-globule transition is known as the phase transition of first (in rigid) or second order (in flexible chains) [1,2]. By following the behavior of the order parameter (degree of “nativeness”) $f_{N}\left(T\right)\in \left[1,0\right]$ or its counterpart (“denaturation” degree) $f_{D}\left(T\right)=1-f_{N}\left(T\right)$, it is possible to describe the phenomenon; the condition:

(1) $$f_{N}\left(T_{D}\right)=f_{D}\left(T_{D}\right)=0.5$$

defines the folding temperature $T_{D}$. If there are no finite size effects or heterogeneity (the account of heteropolymeric effects in the coil-globule transition is outside the scope of the current study), the order parameter at the transition point undergoes an abrupt all-or-none transformation. Responsible for this coil-globule phase transition are strong correlations between repeat unit conformations, which occur due to the van der Waals interactions between the remote repeat units [1]. Changes in external conditions (temperature, pressure, pH, solution composition, etc.) shift the equilibrium in these effective interactions from repulsion (good solvent regime) via neutral conditions (ideal or theta conditions) to attraction (poor solvent regime), which forces the protein to fold. The hydrogen bonds, which are responsible for the formation of secondary structures, have a shorter span and influence the conformations locally. In α-helices, one hydrogen bond fixes the conformations of three subsequent residues [3]. Although the hydrogen bond loops in β-hairpins are roughly twice as long and typically span over five to seven residues [4], the interaction is still local. Therefore, according to the Landau–Peierls theorem [5], such hydrogen bonds cannot per se lead to the coil-globule (phase) transition [1,6]. However, if there are long-range interactions present in the system, the formation of secondary structures can change the effective stiffness of the polypeptide chain, increase stability, and thus, promote the coil-globule transition at equal external conditions. Indirect support for such a mechanism arises from the fact that both the coil-helix transition and protein folding occur at the same interval of external parameters [7]. Thermodynamic cooperativity as a concept is often attributed to the sharpness of the phase transition, which results from the spatially correlated behavior of the particles (in this case, repeating units). The situation of the idealized first order phase transition with correlations that extend throughout the system and lead to the discontinuity of the order parameter corresponds to infinite cooperativity and the zero transition interval. When it comes to the folding of single domain globular proteins of just N<100 repeating units long, the limited system sizes impose constraints onto otherwise infinite correlations at the transition point. Consequently, the folding happens over some small temperature interval ΔT(≠0), which needs to be estimated. Using the Taylor expansion cut at first order, it is possible to approximate the order parameter as $f_{D}^{appr}$ with the help of the tangent at the transition point:

(2) $$f_{D}\left(T\right)\approx f_{D}^{appr}\left(T\right)=f_{D}\left(T_{D}\right)+f_{D}^{\prime }\left(T\right){|}_{T_{D}}\left(T-T_{D}\right).$$

From the definitions of initial and final temperatures as $f_{D}^{appr}\left(T_{1}\right)=0$ and $f_{D}^{appr}\left(T_{2}\right)=1$, one can define the transition interval (see, e.g., [8,9]) as:

(3) $$T_{2}-T_{1}=\Delta T=f_{D}^{\prime }{\left(T\right)|}_{T_{D}}^{-1}.$$

The derivative of the order parameter at the transition point is the experimentally measurable quantity that provides access to information on the system’s cooperativity. The temperature is not the only possible external parameter that can induce the transition. The experiments are often set by changing the concentration of the denaturant such as urea or guanidinium chloride (GdmCl). After repeating the steps behind Equation (2), the resulting expression for the change in the number of bound denaturant molecules during the transition is:

(4) $$n_{2}-n_{1}=\Delta n=f_{D}^{\prime }{\left(n\right)|}_{n_{D}}^{-1},$$

so that the thermodynamic cooperativity of the transition can be still estimated by the measured slope of the transition curve at its middle point.

In this paper, we introduce the protein chain length as a parameter into the two-state model, perform the finite-size scaling of protein folding, and compare the two famous criteria of cooperativity.

## 2. Materials and Methods

The two-state model is the simplest among the folding models, yet is very general and fruitful and therefore deserves a detailed, even pedantic derivation of its well-known formulas, enabling us to trace their origins and limitations. Within the two-state paradigm, the presence of just two possible macroscopic states is assumed: the native globular state with the energy value $E_{N}$ and the denatured coil one with the energy $E_{D}$. To reflect the uniqueness of the native state, a degeneracy $g_{N}=1$ is attributed; a $g_{D}\gg 1$ degeneracy is set for the denatured state to reflect its greater conformational entropic freedom. Without loss of generality, one can assume $E_{N}=0$, $E_{D}\neq 0$ and write down the density of states for the two-state model:

(5) $$g\left(E\right)=\delta \left(E\right)+g_{D}\delta \left(E-E_{D}\right),$$

where δ(x) is the Dirac delta function, resulting in the partition function:

(6) $$Z\left(\beta \right)=\int_{0}^{\infty }dE\;g\left(E\right)\;e^{-\beta E}=1+g_{D}e^{-\beta E_{D}}=\left[N\right]+\left[D\right],$$

where […] is the number of repeat units in the native or denatured state and β=1/T is the inverse temperature. The average energy is just the internal energy of the system and follows directly as:

(7) $$<E\left(\beta \right)>=\frac{\int_{0}^{\infty }dE\;g\left(E\right)\;e^{-\beta E}E}{\int_{0}^{\infty }dE\;g\left(E\right)\;e^{-\beta E}}=-\frac{d\log Z(\beta )}{d\beta }=\frac{g_{D}e^{-\beta E_{D}}}{1+g_{D}e^{-\beta E_{D}}}E_{D},$$

leading to the heat capacity:

(8) $$C_{V}\left(\beta \right)=-\beta^{2}\frac{d<E>}{d\beta }={\left(\beta E_{D}\right)}^{2}\frac{g_{D}e^{-\beta E_{D}}}{{\left(1+g_{D}e^{-\beta E_{D}}\right)}^{2}}.$$

The denaturation degree reads:

(9) $$f_{D}\left(\beta \right)=\frac{\left[D\right]}{\left[N]+[D\right]}=\frac{g_{D}e^{-\beta E_{D}}}{1+g_{D}e^{-\beta E_{D}}}=\frac{<E(\beta )>}{E_{D}}=-\frac{1}{E_{D}}\frac{d\log Z(\beta )}{d\beta },$$

and the equilibrium constant:

(10) $$K_{eq}\left(\beta \right)=\frac{\left[D\right]}{\left[N\right]}=\frac{f_{D}\left(\beta \right)}{1-f_{D}\left(\beta \right)}=g_{D}e^{-\beta E_{D}}.$$

At the transition point, the numbers of repeat units in the native *N* or the denatured *D* state are equal, and with the help of Equation (9), we can express the transition temperature Equation (1) and interval Equation (3) in terms of the two-state model parameters as:

(11) $$T_{D}=\frac{E_{D}}{\log g_{D}}\;;\;\;\Delta T=\frac{4E_{D}}{\log^{2}g_{D}}.$$

The last expression can be rewritten as:

(12) $$E_{D}=\frac{4{T_{D}}^{2}}{\Delta T},$$

resulting in the famous expression for the energetic price of the transition between the two states. Privalov and Kheshinashvili [10] referred to Equation (12) as an approximation, but as we showed above, it is indeed exact within the two-state picture. Since all the above formulae are derived under the assumption of the existence of strictly two states, the results can only be attributed to one cooperative unit, i.e., a part of a molecule that undergoes the transition from *N* to *D* as a whole. Microcalorimetry allows the simultaneous measurement of the transition enthalpies for the whole protein molecule and for the cooperative unit [11]. Potentiometric titration also allows the difference in the degree of ionization to be measured for the entire molecule and compared with the value for the cooperative unit [12].

The order of a conformational transition can be evaluated by analyzing the dependence of the slope of the transition on the molecular weight of the protein (*M*), which is linearly proportional to the degree of polymerization *N*. It is clear that the slope of the phase transition in small systems depends on the dimensions of this system [1,13]. In the case of the first order phase transition, the slope increases proportionally to the number of units in a system [13], while the slope for the second order phase transition is proportional to the square root of this number [1].

The system sizes can be introduced by the reasonable assumption that each repeating unit of the polypeptide chain can be found in one out of Q>2 rotational isomeric states, only one of which corresponds to the native state. Since there is *N* such repeating units, the number of possible states in the denatured conformation for the whole macromolecule and the additive energy of the system read:

(13) $$\begin{array}{c} g_{D}={\left(Q-1\right)}^{N};\;\;E_{D}=\epsilon_{D}N. \end{array}$$

In view of Equation (11), this means:

(14) $$\begin{array}{c} T_{D}\left(N\right)=\frac{\epsilon_{D}}{\log(Q-1)}\;;\;\Delta T\left(N\right)=\frac{4T_{D}}{\log(Q-1)}\frac{1}{N}. \end{array}$$

This is a very interesting result, which shows that within the two-state paradigm, the denaturation temperature does not depend on the system size. Instead, the transition interval is inversely proportional to *N*, which naturally leads to a zero interval at N→∞, just as it should in the case of the phase transition.

## 3. Results and Discussion

The criterion of the two-state cooperativity $k_{2}$ of protein folding has already been discussed in detail (see, e.g., [14,15] and the references therein). It is defined as the ratio of van ’t Hoff and calorimetric enthalpy (energy):

(15) $$k_{2}=\frac{\Delta E_{vH}}{\Delta E_{cal}},$$

where the van ’t Hoff energy is:

(16) $$\Delta E_{vH}=-\frac{d\log K_{eq}\left(\beta \right)}{d\beta }=E_{D},$$

and the amount of heat exchanged during the transition is calculated as the integral under the heat capacity curve:

(17) $$\Delta E_{cal}=\int_{0}^{\infty }dT\;C_{V}\left(T\right)=E_{D}\frac{g_{D}}{1+g_{D}}.$$

According to Equations (13), (16), and (17), the resulting:

(18) $$k_{2}=\frac{\Delta E_{vH}}{\Delta E_{cal}}=1+1/g_{D}=1+O\left(e^{-N\log(Q-1)}\right),$$

is an expression that asymptotically tends to one (from above) for large *N*. It can be concluded that the two-state ansatz, expressed in Equation (5), results in $k_{2}=1$, making it the necessary condition for the transition to be classified as a two-state one. Please note, strictly speaking, that it follows from noting that $k_{2}=1$ means that the transition is a two-state one. In a certain sense, the condition is negative: if $k_{2}$ is different from unity, the transition cannot be a two-state one, while if it is close to unity, it is not enough to conclude the two-state behavior. The folding cooperativity measure:

(19) $$\Omega_{c}=\frac{T_{D}^{2}}{\Delta T}\frac{df_{D}}{dT}{|}_{T=T_{D}}$$

was proposed by Klimov and Thirumalai [16] to compare the cooperativities of different proteins. Based on their collection of experimental and simulation data of protein folding, the same authors later suggested a size scaling law for the folding cooperativity measure $\Omega_{c}\propto N^{\zeta }$ [17], where ζ=1+γ and γ is a susceptibility exponent.

Using our Equation (3), we can significantly simplify the expressions for $\Omega_{c}$. Li et al. defined the interval $\Delta T^{*}=T_{2}^{*}-T_{1}^{*}$ as the width at half-height of the differential curve [17]. One can approximate the peaked curve by a rectangle with sides at $T_{1}^{*}$ and $T_{2}^{*}$ and a height $|f_{D}^{\prime }{\left(T\right)|}_{T_{D}}$ in such a way, that $1=\int_{0}^{\infty }f_{N}^{\prime }\left(T\right)dT\approx {\left|f_{D}^{\prime }\left(T\right)\right|}_{T_{D}}\left(T_{2}^{*}-T_{1}^{*}\right)$. With the account of Equation (3), this leads to the obvious $\Delta T^{*}=\Delta T$, proving that both definitions of the transition interval are equivalent, at least in the sense of asymptotic, size scaling relations. The same Equation (3), when inserted into the cooperativity measure Equation (19), simply results in:

(20) $$\Omega_{c}={\left(\frac{T_{D}}{\Delta T}\right)}^{2}.$$

The result is not surprising, since the $\frac{T_{D}}{\Delta T}$ ratio is common in the studies of finite size effects at phase transitions [18,19,20]. In view of Equation (14), valid for the two-state model, it simply means that:

(21) $$\Omega_{c}={\left(\log g_{D}/4\right)}^{2}\propto N^{2}.$$

**Figure 1:**
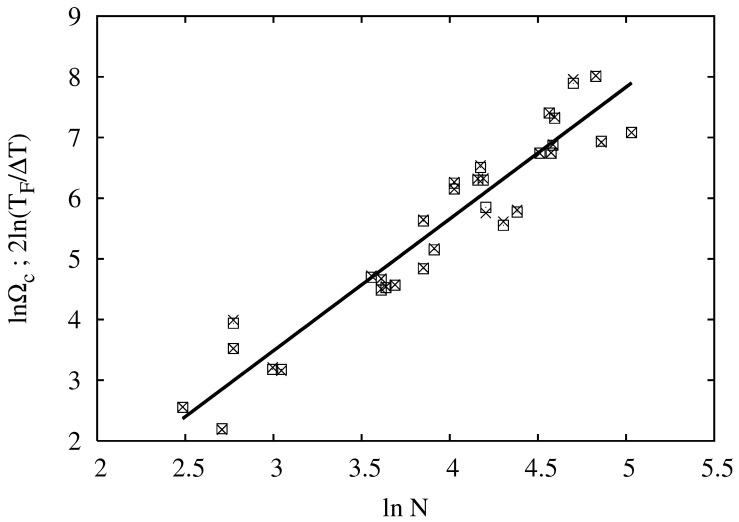
The dependence of $\ln\Omega_{c}$ (×) and $2\ln\left(\frac{T_{D}}{\Delta T}\right)$ (□) vs. lnN. Data taken from the database reported in [17]. (×) correspond to the Klimov and Thirumalai Equation (19), while (□) visualize our result Equation (20). The straight lines, corresponding to the linear fits for both data point collections, are indistinguishable on the graph.

However, if not bound to the two-state paradigm, the more general and model-independent formula expressed with Equation (20) allows establishing direct links between the well-known size scaling relations and the cooperativity measure $\Omega_{c}$. To take into account the possibility for both the first and second order mechanisms of the phase transition, $\frac{T_{D}}{\Delta T}\propto N^{1/d\nu }$ scaling should be considered [18,19,20,21] (instead of $N^{1}$, used by Li et al.), where dν is a critical exponent of the correlation length or radius of gyration; the dν=1 and dν=2 values would correspond to the first and second order phase transition, accordingly. From Equation (20), it immediately follows that:

(22) $$\Omega_{c}\propto N^{2/d\nu },$$

with dν=1 for our case of exactly two-state folders. Klimov, Thirumalai, and Li [16,17] justified the necessity for the new critical exponent ζ=1+γ by fitting the points from their dataset reported in [17] to their expression Equation (19). However, our Equation (22) can be used instead, without invoking the new critical exponent ζ. In order to compare the two approaches, in Figure 1, we re-plot the data from [17] and compare them with our Equation (22). The data points for $\ln\Omega_{c}$ and $2\ln\frac{T_{D}}{\Delta T}$ vs. lnN are superimposed, and the corresponding fitted straight lines are indistinguishable, thus validating Equation (22) over the set of data from [17]. The fit results in $d\nu_{exp}=0.92$, which is close to, but not equal to one. The scaling on the basis of Equation (22) nicely fits the experimental trends and thus allows us to treat protein folding as a true phase transition in a finite system in the sense of Lifshitz–Grosberg–Khokhlov [2]. The fact that the transition interval has the same size scaling exponent as the correlation length is a nice example of the contribution of correlations in protein conformations to folding cooperativity.

There is further experimental evidence that supports our view. Ptitsyn and Uversky proposed the molten globule as the third thermodynamic state of protein molecules in a number of publications [22,23]. Based on the systematic analysis of data on urea and guanidinium chloride induced transition of globular proteins from the native to the unfolded state (N→U), from the native to the molten globule (N→MG) state, and from the molten globule to the unfolded state (MG→U), it has been shown that in all these cases, the cooperativity of unfolding increases linearly with the increase in the molecular weight of the protein up to 25–30 kDa [22,23]. In fact, this cooperativity of all three transitions measured in terms of Δn (see Equation (4)) follows logΔn=dνlogM−b, with $d\nu_{N-U}=0.97$, $d\nu_{N-MG}=1.02$, and $d\nu_{MG-U}=0.89$, all close to the dν=0.92 value, estimated from the temperature inspired set of data from [17]. This means that such a dependence of the cooperativity of urea-induced and guanidinium chloride-induced transitions in small proteins on their molecular weight suggests that all three types of transitions are all-or-none, indicating that the molten globule state is separate from the native and unfolded state by all-or-none transitions [22,23]. Thus, the experimental data on denaturant-induced unfolding of small globular proteins are consistent with the linear $\log\Omega_{c}$ vs. logN dependence described in [17].

The comparison of cooperativity measures shows that each of them has advantages and drawbacks. The strict two-state assumption, expressed in Equation (5) allows the derivation of $k_{2}\approx 1$ at large *N*, which is therefore a necessary condition for the two-state folding. Independent of the chain length, $k_{2}$ allows the statement about which of the proteins under consideration comes closer to the ideal two-state behavior. Instead, in the same N→∞ limit, $\Omega_{c}$ tends to infinity, which means that under other equal conditions, longer chains have higher values of the cooperativity measure $\Omega_{c}$. On the other hand, $k_{2}$, as defined by Equation (15), contains both equilibrium and kinetic quantities, which are only equal when the system has reached equilibrium, and the deviation from unity can be attributed to kinetic traps (see also [15] for the definition and discussion about the kinetic cooperativity). Regarding $\Omega_{c}$, once expressed through $\frac{T_{D}}{\Delta T}$, it becomes a criterion similar to those introduced in other areas of physics to deal with the effects of a finite size at phase transitions. The last fact puts it on very solid grounds.

## 4. Conclusions

In summary, we contribute to a better understanding of the physical basis of the two cooperation criteria under consideration. For the first time, the size scaling expressions for the cooperation criteria are derived and analyzed (Equations (18) and (20)). As a result, we concluded that $k_{2}$ can be conveniently used to compare cooperativity for individual proteins, while $\Omega_{c}$ is more useful for comparing protein folding data sets with respect to size scaling analysis.

## Data Availability

Data Availability Statement: Data sharing not applicable.
